# Bacterial direct-fed microbials fail to reduce methane emissions in primiparous lactating dairy cows

**DOI:** 10.1186/s40104-019-0342-9

**Published:** 2019-05-02

**Authors:** Jeyamalar Jeyanathan, Cécile Martin, Maguy Eugène, Anne Ferlay, Milka Popova, Diego P. Morgavi

**Affiliations:** 1 Université Clermont Auvergne, INRA, VetAgro Sup, UMR 1213 Herbivores, F-63122 Saint-Genès-Champanelle, France; 2 Present address: Laboratory for Animal Nutrition and Animal Product Quality, Faculty of Bioscience Engineering, Ghent University, Coupure Links 653, 9000 Ghent, Belgium

**Keywords:** Bacterial direct-fed microbial, Dairy cow, Methane, Milk fatty acid

## Abstract

Direct-fed microbials (DFM) are considered as a promising technique to improve animal productivity without affecting animal health or harming the environment. The potential of three bacterial DFM to reduce methane (CH_4_) emissions, modulate ruminal fermentation, milk production and composition of primiparous dairy cows was examined in this study. As previous reports have shown that DFM respond differently to different diets, two contrasting diets were used in this study. Eight lactating primiparous cows were randomly divided into two groups that were fed a corn silage-based, high-starch diet (HSD) or a grass silage-based, high-fiber diet (HFD). Cows in each dietary group were randomly assigned to four treatments in a 4 × 4 Latin square design. The bacterial DFM used were selected for their proven CH_4_-reducing effect *in vitro*. Treatments included control (without DFM) and 3 DFM treatments: *Propionibacterium freudenreichii* 53-W (2.9 × 10^10^ colony forming units (CFU)/cow per day), *Lactobacillus pentosus* D31 (3.6 × 10^11^ CFU/cow per day) and *Lactobacillus bulgaricus* D1 (4.6 × 10^10^ CFU/cow per day). Each experimental period included 4 weeks of treatment and 1 week of wash-out, with measures performed in the fourth week of the treatment period. Enteric CH_4_ emissions were measured during 3 consecutive days using respiration chambers. Rumen samples were collected for ruminal fermentation parameters and quantitative microbial analyses. Milk samples were collected for composition analysis. Body weight of cows were recorded at the end of each treatment period. Irrespective of diet, no mitigating effect of DFM was observed on CH_4_ emissions in dairy cows. In contrast, *Propionibacterium* increased CH_4_ intensity by 27% (g CH_4_/kg milk) in cows fed HSD. There was no effect of DFM on other fermentation parameters and on bacterial, archaeal and protozoal numbers. Similarly, the effect of DFM on milk fatty acid composition was negligible. *Propionibacterium* and *L. pentosus* DFM tended to increase body weight gain with HSD. We conclude that, contrary to the effect previously observed *in vitro*, bacterial DFM *Propionibacterium freudenreichii* 53-W, *Lactobacillus pentosus* D31 and *Lactobacillus bulgaricus* D1 did not alter ruminal fermentation and failed to reduce CH_4_ emissions in lactating primiparous cows fed high-starch or high-fiber diets.

**Electronic supplementary material:**

The online version of this article (10.1186/s40104-019-0342-9) contains supplementary material, which is available to authorized users.

## Background

Livestock farming is considered a major contributor to anthropogenic methane (CH_4_) emissions, which is mainly attributed to ruminants [1]. Methane production is also energetically wasteful for ruminants resulting in a loss of 2–12% of the ingested feed energy [2]. Several dietary strategies based on additives and supplements have been proposed to mitigate rumen methanogenesis but only few of them have shown persistent effect *in vivo* without negative impacts to the host animal and the environment [3, 4]. Use of direct-fed microbials (DFM) is one possible option that could be sustainable and easily acceptable by both consumers and producers [5].

Direct-fed microbials are used in the dairy sector to improve animal productivity and health [6, 7]. *Propionibacterium* and *Lactobacillus* spp. alone or in combination are the most common bacterial DFM used in ruminant production [7]. A metabolic aspect that characterizes these bacterial species is the production of propionate, which is a H_2_-consuming reaction [8]. Promoting this pathway is expected to produce less H_2_ and consequently less CH_4_ in the rumen. However, *in vivo* studies using *Propionibacterium* and/or *Lactobacillus* spp. as modulators of enteric CH_4_ production showed contrasting results with decreases, no effect or even increases in CH_4_ emissions [4, 9–11]. These differences could be originated from several factors such as type of ruminant, physiological stage, and diet, but also due to differences in the strains of DFM used.

The bacterial DFM used in this study: *Propionibacterium freudenreichii* 53-W, *Lactobacillus pentosus* D31 and *Lactobacillus bulgaricus* D1 were previously selected for their CH_4_-decreasing effect *in vitro* [9]. They were also tested in adult wethers fed a hay-based diet (70% natural grassland hay and 30% concentrate) at maintenance with contrasting results [9]. Whereas *L. pentosus* reduced CH_4_ emissions (g/kg DMI), no effect was observed for *L. bulgaricus* and *P. freudenreichii* increased CH_4_ emissions (g/kg DMI). The efficacy of DFM may differ depending on the animal species, physiological stage and diet [4, 10–12]. The objective of this study was to examine the potential of three selected bacterial DFM to modulate ruminal fermentation in lactating primiparous cows. The effect on milk production and composition, more particularly fatty acid (FA) composition, was also monitored. As efficacy of bacterial DFM has been shown to be affected by diet a high-starch diet (HSD) and a high-fiber diet (HFD) were used in this study.

## Methods

This study was conducted using the animal facilities at the French National Institute for Agricultural Research (INRA) in Theix. Procedures on animals used in this study complied with the guidelines for animal research of the French Ministry of Agriculture and all other applicable National and European guidelines and regulations.

### Animals, experimental design, and diets

Eight lactating primiparous Holstein cows (age of 2.9 ± 0.4 years, mean ± SD) were housed in individual stalls during the study. The cows were randomly allocated into two balanced groups of four animals and fed two different basal diets: one based on corn silage, hereafter called high-starch diet (HSD), and the second based on grass silage, hereafter called high-fiber diet (HFD; Table 1). At the start of the study, average daily milk production was 22.8 ± 4.9 and 22.6 ± 1.1 kg/cow, days in milk 83.2 ± 11.3 and 91 ± 15.6 days, and body weight 587.5 ± 51.1 and 585.7 ± 32.3 kg for cows fed HSD and HFD, respectively.

**Table 1:** Ingredients and chemical composition of the high-starch and high-fiber control diets used in this study

Items	Control diets^a^	
	High-starch diet	High-fiber diet
Ingredients, % of DM		
Corn silage	44.0	_^b^
Grass silage	_	55.0
Hay	11.0	_
Grain mix^c^	34.2	_
Citrus pulp	_	12.0
Dehydrated beet pulp	_	20.0
Molasses, beet	_	5.0
Soybean meal	8.7	8.0
Urea	1.0	_
Cane molasses	1.1	_
Chemical composition, % of DM		
OM	92.2	85.1
CP	12.5	12.2
NDF	35.4	48.4
ADF	19.5	29.3
Starch	27.4	1.8
Ether extract	2.3	2.3
Fatty acids (FA), g/100 g of total FA		
12:0	0.17	0.34
14:0	0.35	0.89
16:0	20.7	21.7
*cis*-9 16:1	0.72	2.03
18:0	2.57	2.13
*cis*-9 18:1	19.4	9.8
18:2*n*-6	44.9	28.5
18:3*n*-3	7.3	28.0
GE, MJ/kg DM	16.8	16.9

^a^Each cow was fed 250 g mineral mix comprising (g/kg): P, 2.5; Ca, 20; Mg, 4.5; Na, 3.5 (Galaphos Midi Duo GR, CCPA, Aurillac, France)

^b^Ingredients not included

^c^Composition: barley (14.1% of DM), wheat (10.9% of DM) and corn (9.2% of DM)

Cows in each group were randomly assigned to four treatments in a 4 × 4 Latin square design that were run in parallel. The treatments were 1) Control without DFM (CTL), 2) *Propionibacterium freudenreichii* 53-W (2.9 × 10^10^ colony forming units (CFU)/cow per day), 3) *Lactobacillus pentosus* D31 (3.6 × 10^11^ CFU/cow per day) and 4) *Lactobacillus bulgaricus* D1 (4.6 × 10^10^ CFU/cow per day). The dose of each DFM (CFU/mL rumen fluid) was chosen considering cost of production and the results from an earlier study with the same DFM preparations administered to sheep fed a hay-based diet [9]. *Propionibacterium freudenreichii* 53-W (DSM 20271) was obtained from DSMZ (Deutsche Sammlung von Mikroorganismen und Zellkulturen GmbH, Braunschweig, Germany) and both *Lactobacillus* species were obtained from Danone culture collection (Danone Research, Palaiseau, France). The DFM preparations used in this study were obtained from Danone Research (Palaiseau, France) in a frozen pellet form. Their viability was checked prior to the study. Weighed pellets were thawed in 0.1% sterile peptone solution, serially diluted and inoculated onto agar plates (DSMZ medium 91 for *P. freudenreichii* and MRS medium for both *Lactobacillus* species). Plates were incubated at 39 °C for 48 h before colony counts. Results were in agreement with the quantity of CFU stated by the manufacturer.

Diets were formulated at the beginning of the study to meet the energy and protein requirements for maintenance and lactation of dairy cows based on INRA nutritional recommendation for ruminants [13]. Diets were free from antibiotics, chemical buffer and yeast to avoid potential interfering effect with the effect of bacterial DFM tested in this study. Two weeks before starting the study, cows in both groups were fed CTL diet ad libitum. Then, throughout the study, feeds were restricted to 90% of their ad libitum intake to ensure complete consumption of the diet. Each experimental period (5 weeks) consisted of 4 weeks of treatment and 1 week of washout, without DFM supplementation. Cows were fed twice daily with 60% of the daily ration at 07:00 h and 40% at 16:00 h. During the treatment period, DFM preparations were administered during the morning feeding. Each day, just before feed distribution, the appropriate amount of pellets were thawed in 50 mL of 0.1% sterile peptone solution at room temperature. To ensure the entire DFM consumption, the 50-mL doses were mixed with a small portion of silage (about 500 g sampled from their diet) and offered before feeding. The amounts of feed offered and refused were weighed daily to estimate DMI. Cows were allowed continuous access to water and water intake was measured for each cow. The body weight of each animal was recorded at the end of each experimental period, 3 h after morning feeding.

### Measurements and analyses

#### Feed analysis

The dry matter content of each feed ingredient was determined (103 °C for 24 h, ISO 6496 [14]) weekly for hay and concentrates and twice per week for silages throughout the experimental period. During the last week of each experimental period (week 4), silage, hay and concentrates were sampled (about 100 g) daily and were pooled at the end of the week. Samples of silage were stored at − 20 °C and samples of hay and concentrates were stored at 4 °C. At the end of the study, all feed samples were dried in an oven and ground (1-mm screen) before chemical analyses (InVivo Labs, Saint Nolff, France). Organic matter was determined by ashing samples at 550 °C for 6 h (method 942.05; [15]). Fiber (NDF and ADF) was determined by sequential procedures [16] after pre-treatment with amylase and expressed exclusive of residual ash. Total N was analyzed by combustion according to the Dumas method (method 968.06; [15]) and CP content was calculated as N × 6.25. Ether extract was determined after acid hydrolysis (method 954.02; [15]). Starch content was analyzed using an enzymatic method [17]. Briefly, samples are incubated in a shaking water bath with pancreatic α-amylase and amyloglucosidase for 16 h at 37 °C, during which starch is hydrolyzed to *D*-glucose by the combined action of the enzymes. Then, the *D*-glucose is measured with glucose oxidase/peroxidase reagent. The gross energy (GE) was analyzed by isoperibolic calorimetry (C200 model; IKA, Staufen, Germany).

#### Enteric methane

In the last week of the experimental period (week 4, days 2–4) enteric CH_4_ emission was determined using individual open circuit respiration chambers (1 cow/chamber) for 3 consecutive days as described in Guyader et al. [18]. Cows were allocated to the same chamber so that the DFM effect was not confounded with the chamber effect. Air leaks from the chambers were examined before the start of the experiment using water-based smoke machines (Kool Light-FOGGER 1500E; EPICAP, Saint-Symphorien d’Ozon, France). The chambers operated at a slightly negative pressure, with an air flow averaging 743.6 ± 19.61, 792.1 ± 17.89, 771.7 ± 14.40 and 756.6 ± 18.43 m^3^/h for periods 1, 2, 3 and 4 respectively. Continuous air sampling was performed in each chamber at a 0.1-Hz sample frequency for 5 min every 25 min and analyzed for CH_4_ gas concentrations with an infrared gas analyzer (Ultramat 6, Siemens, Karlsruhe, Germany). The chambers were opened twice daily at 07:00 h and 15:00 h for about 20 min for milking and subsequent feeding. The gas analyser was calibrated at the start of every CH_4_ measurement period with pure N_2_ and a certified standard gas mixture of CO_2_ (1.36 g/m^3^) and CH_4_ (0.459 g/m^3^). Real time gas emissions in a chamber were calculated by the difference between chamber and ambient gas concentrations multiplied by the airflow corrected for temperature, relative humidity, and pressure according to the Wexler equation [19]. Calculations of CH_4_ yield (g CH_4_/kg DMI) and intensity (g CH_4_/kg milk) were done using data on DMI and milk production when cows were in chambers.

#### Ruminal fermentation and microbes

In the last week of the experimental period (week 4) rumen samples (approximately 500 mL) were collected 3 h after the morning feeding for two non-consecutive days (day 1 and 5) using a stomach tube fitted with a vacuum pump. The samples were subjected to visual examination to ensure that they were not contaminated with saliva. Values of pH were also used as an additional control. Samples suspected to be contaminated were removed, and fresh samples were taken.

The pH of each sample was recorded immediately with a portable pH-meter (CG840, electrode Ag/AgCl, Schott Gerate, Hofhein, Germany). One aliquot of rumen contents (about 200 mL) was strained through a polyester monofilament fabric (mesh size 250 μm) and the filtrate was sampled for analysis of VFA, ammonia-N (NH_3_-N), and protozoa counts. Samples for VFA were prepared by transferring 0.8 mL filtrate into a micro-tube containing 0.5 mL of a crotonic-metaphosphoric acid solution (crotonic acid 0.4% *wt*/*vol*, metaphosphoric acid 2% *wt*/*vol*, in HCl 0.5 mol/L) and stored at − 20 °C until analysis. For NH_3_-N, 1 mL of rumen filtrate was mixed with 0.1 mL of 5% H_3_PO_4_ and stored at − 20 °C until analysis. For protozoa counts, 2 mL of the rumen filtrate was mixed with 2 mL of methyl-green-formalin and saline solution (MFS) and preserved from light until counting. For quantitative microbial analysis, another aliquot (about 200 mL) of rumen contents was frozen immediately at − 80 °C and subsequently lyophilized. Lyophilized samples were then ground and stored at − 80 °C until DNA was extracted. For each sampling time, unfiltered rumen contents were dried at 103 °C for 24 h for DM determination.

Volatile fatty acid concentrations were determined by gas chromatography [20] on a Perkin-Elmer Clarus 580 GC (Perkin Elmer, Courtaboeuf, France) equipped with a column Stabilwax – DA (30 m × 0.53 mm i.d.) and using crotonic acid as the internal standard. The concentration of NH_3_-N in rumen fluid was determined using the Berthelot reaction [21]. Rumen fluid/MFS solutions were diluted in an equal volume of phosphate buffer saline solution (PBS) and protozoa were enumerated in a Neubaeur chamber [22].

Total genomic DNA was extracted from ground lyophilized rumen samples using a bead beating and column purification (QIAamp DNA stool mini kit, Qiagen, Valencia, CA) method [23]. The yield and purity of the extracted DNA was determined using a NanoDrop spectrophotometer (Thermo Scientific, Wilmington, DE) and stored at − 20 °C. The primers used in this study are listed in Additional file 1: Table S1.

Quantitative real-time PCR assays were performed on a StepOne™ system (Applied Biosystems, Courtabeuf, France) using SYBR Ex Taq™ pre mixture (Takara Bio Inc., Otsu, Japan). Amplification of 16S rRNA genes of *P. freudenreichii* and *L. bulgaricus*, and intergenic spacer regions (16S–23S) of *L. pentosus* were performed as described in Jeyanathan et al. [9]. Quantification of bacterial 16S rRNA and methanogenic *mcrA* genes were performed as previously described [24].

#### Milk production and composition

Cows were milked twice daily at 07:00 h and 15:00 h, and milk production of individual animals was recorded electronically throughout the study except for the last week of treatment period (week 4) when cows were in chambers. In week 4, milking and weighing were done manually. Samples of milk for the measurement of fat, protein, and lactose were collected individually once per week and treated with preservative (bronopol-B2; Trillaud, Surgeres, France). Samples of unpreserved milk were also collected at each milking over 2 non-consecutive days (Tuesday and Thursday) of week 4 of the experimental period and stored at − 20 °C until analysis for FA composition.

Milk fat and protein contents were determined by mid-infrared spectrophotometry using a Milkoscan 4000 (Foss Electric, Hillerød, Denmark). The FA of the lyophilized milk samples were methylated and analyzed as before [25] with some modifications: 2 mL of 0.5 mol/L sodium methanolate and 1 mL hexane were mixed with the lyophilised milk at 50 °C for 15 min, followed by the addition of 1 mL 12 mol/L HCl 5% in methanol (*v*/*v*) at 50 °C for 15 min. The fatty acid methyl esters (FAME) were washed with a saturated K_2_CO_3_ solution and recovered with 1.5 mL hexane. The FAME were injected (0.6 μL) by auto-sampler into a gas chromatograph equipped with a flame ionisation detector (Agilent Technologies 7890A, Wilmington, USA) and separated on a 100 m × 0.25 mm i.d. fused-silica capillary column (CP-Sil 88, Chrompack, Middelburg, The Netherlands). A reference standard butter (CRM 164, Commission of the European Communities, Community Bureau of Reference, Brussels, Belgium) was used to estimate correction factors for short-chain FA (C4:0 to C10:0). Identification of FAME was accomplished by comparison to a standard mixture purchased from Nu-Chek-Prep, Inc. (Elysian, MN 56028 USA). Mixtures of *cis*/*trans* (9–12) isomers of linoleic acid methyl ester and *cis* and *trans* (9–11) and (10–12) isomers of CLA methyl esters purchased from Sigma-Aldrich Corporation (38297 Saint Quentin Fallavier, France) were used for complete identification.

### Statistical analysis

Data were averaged per period and per animal and analyzed using the Mixed procedure of SAS version 9.4 (SAS Institute, 2004). Data from HSD and HFD were analyzed separately as comparison between diets was not the objective of the study. The following model was used: Y_*ijk*_ = μ + T_*i*_ + P_*j*_ + C_*k*_ + e_*ijk*_*, w*here: Y_*ijk*_ are observations for dependent variables; μ is the overall mean; T_*i*_ is the fixed effect of DFM (control, *Propionibacterium*, *L. pentosus* and *L. bulgaricus*); P_*j*_, is the fixed effect of period (*j* = 1–4); C_*k*_ is the random effect of cow; and e_*ijk*_ is the random residual error. The effect of individual DFM supplementation was tested using Dunnett’s test, whereas orthogonal contrasts were performed to evaluate the effect of CTL versus all DFM treatments. Data were considered significant at *P* < 0.05, and trends were discussed at 0.05 < *P* ≤ 0.10.

## Results and discussion

In this study, we tested the effects of bacterial DFM on enteric CH_4_, ruminal fermentation parameters, milk production and composition and the quantity of ruminal microbes in lactating primiparous dairy cows fed two contrasting diets differing in starch and fiber contents. Differences induced by diets (shown in Tables 2, 3 and supplementary Tables) were as expected for diets of similar composition [10] and are not further discussed as they were not the aim of the study. Additionally, the effects of these type of diets on ruminal fermentation and production are well documented [10].

**Table 2:** Enteric methane (CH_4_) emissions of lactating cows fed high-starch or high-fiber diets (CTL) supplemented with bacterial direct-fed microbials (DFM) *Propionibacterium freudenreichii* 53 W (PF), *Lactobacillus pentosus* D31 (LP), and *Lactobacillus bulgaricus* D1 (LB)

CH_4_ emissions	Treatment				SEM^a^	*P*-value	
	CTL	PF	LP	LB		Treatment	CTL vs DFM^b^
CH_4_, g/d							
High-starch diet	286.4	327.8	303.9	271.4	21.12	0.33	0.56
High-fiber diet	290.8	310.0	301.4	292.3	9.79	0.51	0.38
CH_4_, g/kg DMI							
High-starch diet	20.0	22.8	21.3	18.7	2.19	0.62	0.72
High-fiber diet	23.9	24.8	24.0	24.0	1.16	0.93	0.81
CH_4_, g/kg milk							
High-starch diet	13.1	16.7^*^	14.6	12.6	0.78	0.02	0.12
High-fiber diet	18.9	18.7	18.2	19.0	1.53	0.98	0.87
CH_4_, g/kg ECM^c^							
High-starch diet	12.6	15.6	14.4	12.5	1.02	0.15	0.22
High-fiber diet	18.1	17.4	17.0	18.9	1.11	0.64	0.80

^a^SEM-standard error of the means

^b^*P*-value for control vs all direct-fed microbials (DFM) within each diet

^c^ECM-energy corrected milk [(0.327 × kg of milk) + (12.95 × kg of fat) + (7.65 × kg of protein)]

^*^Significantly (*P* ≤ 0.05) different from CTL group

**Table 3:** Intake, milk production, milk composition and body weight (BW) gain of lactating cows fed high-starch or high-fiber diets (CTL) supplemented with bacterial direct-fed microbials (DFM) *Propionibacterium freudenreichii* 53 W (PF)*, Lactobacillus pentosus* D31 (LP), and *Lactobacillus bulgaricus* D1(LB)

Items	Treatment				SEM^a^	*P*-value	
	CTL	PF	LP	LB		Treatment	CTL vs DFM^b^
Dry matter intake, kg/d							
High-starch diet	14.3	14.5	14.5	14.6	0.69	0.99	0.80
High-fiber diet	12.2	12.5	12.6	12.3	0.72	0.97	0.74
Water intake, L/d							
High-starch diet	53.1	62.9	62.0	59.2	5.78	0.65	0.25
High-fiber diet	56.5	50.7	47.5	51.6	3.25	0.33	0.11
Milk, kg/d							
High-starch diet	22.1	19.7	20.9	21.6	0.79	0.23	0.18
High-fiber diet	16.3	16.9	17.0	15.6	1.40	0.89	0.90
Fat, g/kg milk							
High-starch diet	37.0	40.1	36.1	36.1	2.57	0.66	0.88
High-fiber diet	38.7	41.4	41.1	37.7	2.85	0.75	0.68
Protein, g/kg milk							
High-starch diet	29.7	30.6	30.1	29.9	2.10	0.99	0.83
High-fiber diet	27.1	27.8	27.9	26.3	1.38	0.82	0.91
ECM^c^, kg/d							
High-starch diet	22.7	21.2	21.3	22.1	0.70	0.41	0.17
High-fiber diet	16.4	17.9	18.0	15.8	1.16	0.47	0.55
Efficiency^d^,							
High-starch diet	1.60	1.46	1.48	1.52	0.08	0.65	0.27
High-fiber diet	1.35	1.45	1.42	1.28	0.05	0.20	0.62
Body weight gain, kg							
High-starch diet	−6.0	11.7^¶^	13.0^¶^	5.7	4.98	0.08	0.02
High-fiber diet	−3.0	4.2	0.5	−3.2	7.85	0.89	0.71

^a^SEM-standard error of the means

^b^*P*-value for control vs all direct-fed microbials (DFM) within each diet

^c^ECM-energy corrected milk [(0.327 × kg of milk) + (12.95 × kg of fat) + (7.65 × kg of protein)]

^d^Efficiency = ECM/Dry matter intake

^¶^0.05 < *P* ≤ 0.10 from CTL group

### Enteric methane and ruminal fermentation

Cows supplemented with *Propionibacterium* numerically emitted more CH_4_ than CTL particularly with HSD (Table 2). When calculated as CH_4_ intensity expressed in g/kg milk, *Propionibacterium* increased emission by 27% (*P* < 0.05). Supplementation of *L. bulgaricus* or *L. pentosus* did not affect daily CH_4_ emission (g/d), yield or intensity (*P* > 0.05). Concentrations of total VFA and NH_3_-N, and VFA profile were similar among DFM treatments for both diets (Additional file 2: Table S2).

Previous studies have shown that the effect of bacterial DFM in the rumen can vary depending on the type of DFM strain, physiological conditions of the animal [26], and composition of diet [4, 10, 11]. In studies using *Propionibacterium acidipropionici* strains P169 and P5 and *Propionibacterium jensenii* P54, reduced CH_4_ emissions (g CH_4_/kg DMI) were reported in beef steers fed a high-forage diet [4], whereas the same strains failed to show any effect on beef heifers fed a high-grain diet [11]. A similar observation was reported by Philippeau et al. [10] using a combination of *P. jensenii* and *Lactobacillus plantarum*. The combined DFM decreased CH_4_/kg DMI in lactating cows fed low starch diet but was ineffective with a high starch diet. It was suggested that the efficacy of *Propionibacteria* to increase propionate levels in the rumen and subsequently reduce CH_4_ emissions might not be observed with high-grain diets where propionate concentration is naturally high [11]. The increases in CH_4_ emissions observed with the supplementation of *P. freudenreichii* 53-W in our study cannot be explained by the above hypothesis as, as mentioned above, there were no changes in VFA profiles (Additional file 2: Table S2). However, in our previous study with wethers, this strain also increased CH_4_ emissions (g CH_4_/kg DMI) [5] and a similar observation (increased trend in g CH_4_/kg DMI) was reported by Vyas et al. [27] in beef heifers fed a mixed diet (60:40 forage to concentrate ratio on DM basis) with *Propionibacterium* supplementation (*P. freudenreichii* T114, T54 and *P. thoenii* T159). In the present study, the starch level of HSD was similar to the study of Vyas et al. [27]. This can partly explain the similar results between these studies.

**Fig. 1:**
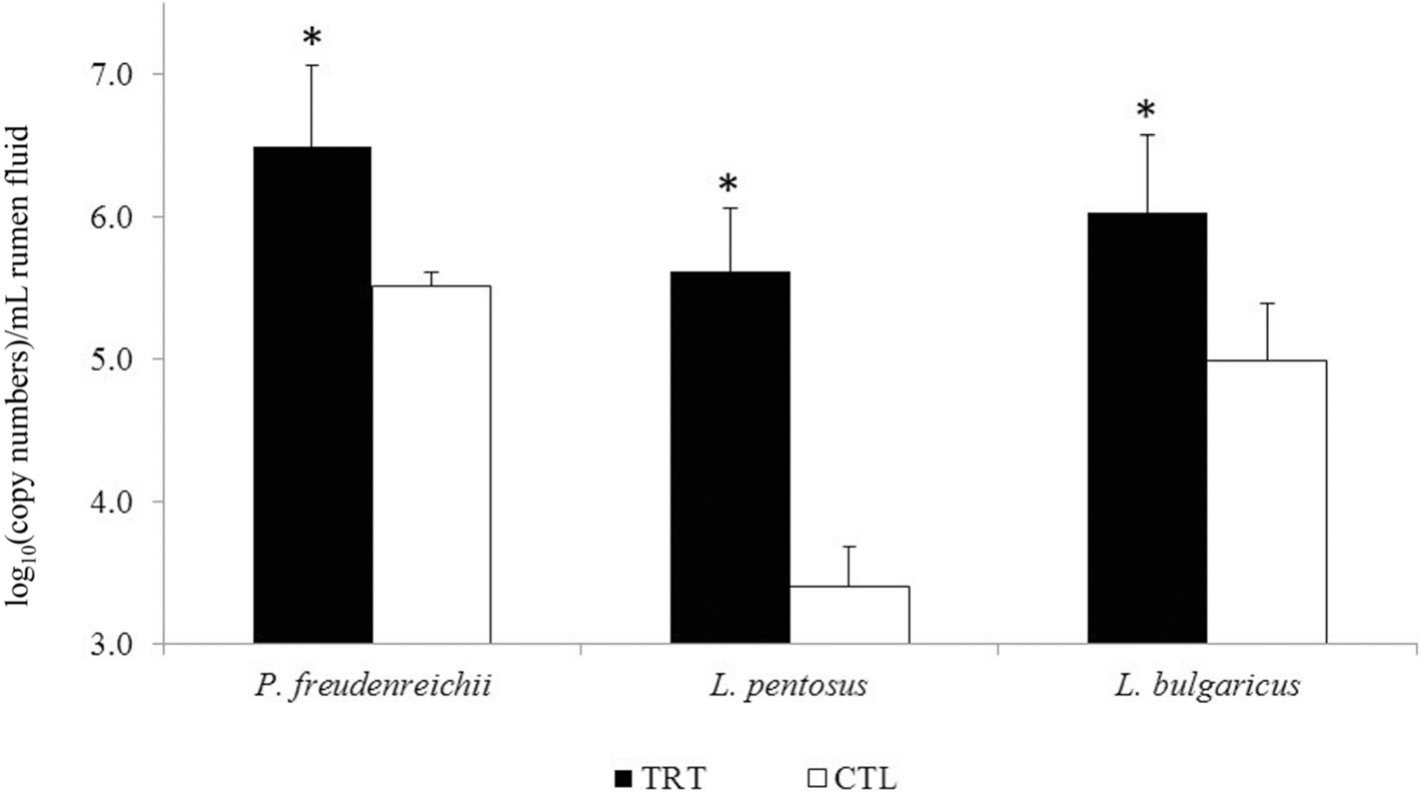
Average abundance of 16S rRNA copies of *Propionibacterium freudenreichii* (PF) and *Lactobacillus bulgaricus*, and 16S–23S intergenic region copies of *Lactobacillus pentosus* in the rumen of dairy cows fed high-starch (HSD) and high-fiber diets (HFD), collected 3 h after administration of direct-fed microbials. CTL-Control cows (in white), TRT-DFM treated cows (in black). Please note that *Y* axis starts at 3 and not 0. * significantly (*P* ≤ 0.05) different from CTL group

A possible reason of DFM failure is that added bacteria were not active or not present in sufficient numbers to have a detectable effect. The viability of the bacterial DFM inocula was tested before utilization and their presence was assessed in the rumen 3 h after administration. The abundance of all three DFM 3 h after administration was higher (tenfold or more) when compared to CTL cows (*P* < 0.05; Fig. 1). However, it can not be excluded that these concentrations were not high enough to modulate ruminal functions. The doses used for the three DFM was chosen based on our previous study in wethers [9] and also for practical considerations of industrial production. These doses are comparable and rather in the high end of the range found in the literature [4, 10, 11, 27, 28]. For the effect of DFM supplementation on the numbers of other ruminal microbial groups, there was no effect on 16S rRNA copy numbers of total bacteria and *mcrA* copy numbers of total methanogens. Similarly, no treatment effect was observed in total protozoal counts or protozoal profile (Additional file 3: Table S3).

### Dry matter intake, milk production and composition

DFM supplementation did not influence DMI, milk production or protein and fat yields (Table 3). Improvements in milk production (4.6%) were reported in multiparous dairy cows (with 3 or more lactations) fed a high-grain diet supplemented with *Propionibacterum* strain P169 [28]. In the same study no difference in milk production was observed with *Propionibacterium* supplementation in younger dairy cows (up to 2 lactations). A similar finding on milk production was reported in dairy cows fed a total mixed ration supplemented with *Propionibacterium* strain P169 [26], in which the positive effect of the DFM was more marked in multiparous than in primiparous cows. The studies cited above suggest that parity may have an influence on the response to DFM, with primiparous cows, like the ones used in our study, being less reactive. More *in vivo* studies needed to confirm this suggestion. Notwithstanding, strain particularities and other factors might also be involved.

Although we did not find any effect of individual DFM on milk performance, BW increased when HSD was supplemented with DFM (*P* < 0.05; Table 3). These changes in BW were mainly driven by *Propionibacterium* and *L. pentosus*. Although not statistically significant, a similar numerical trend in BW was observed when HFD was supplemented with DFM. Improved energy balance and increased BW in *Propionibacterium*-treated cows were observed previously by Francisco et al. [29] in early lactation cows. In our study, the restriction of DMI to 90% of the ad libitum intake may have exacerbated a potential influence of *Propionibacterium* and *L. pentosus* on the energy balance and partitioning in cows fed HSD. Numerically lower milk production in cows fed HSD with *Propionibacterium* supplementation resulted in increased CH_4_ intensity expressed in g/kg milk (*P* < 0.05). The metabolic shift that may have been induced by these bacterial DFM could be due to the physiological status of primiparous dairy cows that mobilize significantly less body reserves than second- and third-parity cows [30]. This mode of action beyond the gastrointestinal tract should be further explored using a larger number of both primiparous and multiparous lactating cows.

### Milk fatty acid composition

Milk fatty acids were determined because they can be used as proxies to estimate CH_4_ emissions [31]. Also, several strains of *Propionibacterium* and *Lactobacillus* species have been identified as potential producers of conjugated linoleic acids (CLA) [32]. In this study, the milk FA composition was affected by diet as expected (statistics not presented) but DFM induced almost no effect (Additional file 4: Table S4).Additional file 4: Table S4 shows also some other minor changes that were particularly detected using orthogonal contrasts.

Apas et al. [33] showed that supplementation of a mixture of *Enterococcus, Lactobacillus* and *Bifidobacterium* strains modified milk FA composition of goats with increases in *cis*-9, *trans*-11 CLA content. In contrast, we did not see any changes in milk *cis*-9, *trans*-11 CLA concentration due to DFM supplementation. The absence of clear changes in the FA profile of milk is in line with other observations.

## Conclusions

The bacterial DFM *P. freudenreichii* 53-W increased CH_4_ emissions intensity (g CH_4_/kg milk) when cows were fed a high starch diet, whereas, none of the DFM used (*P. freudenreichii* 53-W, *L. pentosus* D31 or *L. bulgaricus* D1) affected ruminal fermentation and production parameters in lactating primiparous dairy cows irrespective of diet.

Most information on the effect of DFM on ruminal fermentation and CH_4_ reduction has been obtained* in vitro*. The results of this work should be taken as a cautionary note as bacteria selected for their modulating activities *in vitro* were not able to induce similar effects *in vivo* and for one DFM the opposite effect was observed for CH_4_ emission. Although discrepancy between *in vitro* and *in vivo* studies is generally known, published studies on this aspect are scarce. Reporting these kinds of studies, where the original hypothesis was not supported by the results, is necessary for an unbiased body of information. To explain this discrepancy, it is important that in future work, strains should be clearly identified, and doses and mode of administration stated.

## Additional files

Additional file 1: **Table S1.** Primers used in this study. (DOCX 33 kb)

Additional file 2: **Table S2.** Ruminal fermentation parameters of lactating cows fed high-starch (HSD) or high-fiber diets (HFD) supplemented with bacterial direct-fed microbials (DFM) *Propionibacterium freudenreichii* 53 W (PF), *Lactobacillus pentosus* D31 (LP), and *Lactobacillus bulgaricus* D1 (LB). (DOCX 33 kb)

Additional file 3: **Table S3.** Ruminal concentration of bacteria, archaea, and protozoa (per mL rumen fluid) of lactating cows fed high-starch (HSD) or high-fiber diets (HFD) supplemented with bacterial direct-fed microbials (DFM) *Propionibacterium freudenreichii* 53 W (PF), *Lactobacillus pentosus* D31 (LP), and *Lactobacillus bulgaricus* D1 (LB). (DOCX 31 kb)

Additional file 4: **Table S4.** Major milk fatty acid (FA) composition of cows fed high-starch (HSD) or high-fiber diets (HFD) supplemented with bacterial direct-fed microbials (DFM) *Propionibacterium freudenreichii* 53 W (PF), *Lactobacillus pentosus* D31 (LP), and *Lactobacillus bulgaricus* D1 (LB). (DOCX 69 kb)

## Abbreviations

ADF: Acid detergent fibre
CFU: Colony forming units
CP: Crude protein
CTL: Control without DFM
DFM: Direct-fed microbials
DMI: Dry matter intake
FA: Fatty acid
FAME: Fatty acid methyl esters
GE: Gross energy
h: Hour
HFD: High-fibre diet
HSD: High-starch diet
NDF: Neutral detergent fibre

## References

[1] Gerber PJ, Steinfeld H, Henderson B, Mottet A, Opio C, Dijkman J (2013). Tackling climate change through livestock – A global assessment of emissions and mitigation opportunities.

[2] Johnson KA, Johnson DE (1995). Methane emissions from cattle. J Anim Sci.

[3] Hristov AN, Oh J, Giallongo F, Frederick TW, Harper MT, Weeks HL, et al. An inhibitor persistently decreased enteric methane emission from dairy cows with no negative effect on milk production. Proc Natl Acad Sci USA. 2015. 10.1073/pnas.1504124112 PMC4553761 26229078

[4] Vyas D, McGeough EJ, McGinn SM, McAllister TA, Beauchemin KA (2014). Effect of *Propionibacterium* spp. on ruminal fermentation, nutrient digestibility, and methane emissions in beef heifers fed a high-forage diet. J Anim Sci.

[5] Jeyanathan J, Martin C, Morgavi DP (2014). The use of direct-fed microbials for mitigation of ruminant methane emissions: a review. Animal.

[6] Adams MC, Luo J, Rayward D, King S, Gibson R, Moghaddam GH (2008). Selection of a novel direct-fed microbial to enhance weight gain in intensively reared calves. Anim Feed Sci Tech.

[7] McAllister TA, Beauchemin KA, Alazzeh AY, Baah J, Teather RM, Stanford K (2011). Review: the use of direct fed microbials to mitigate pathogens and enhance production in cattle. Can J Anim Sci.

[8] Marvin-Sikkema FD, Richardson AJ, Stewart CS, Gottschal JC, Prins RA (1990). Influence of hydrogen-consuming bacteria on cellulose degradation by anaerobic fungi. Appl Environ Microbiol.

[9] Jeyanathan J, Martin C, Morgavi DP (2016). Screening of bacterial direct-fed microbials for their antimethanogenic potential in vitro and assessment of their effect on ruminal fermentation and microbial profiles in sheep. J Anim Sci.

[10] Philippeau C, Lettat A, Martin C, Silberberg M, Morgavi DP, Ferlay A (2017). Effects of bacterial direct-fed microbials on ruminal characteristics, methane emission, and milk fatty acid composition in cows fed high- or low-starch diets. J Dairy Sci.

[11] Vyas D, McGeough EJ, Mohammed R, McGinn SM, McAllister TA, Beauchemin KA (2014). Effects of *Propionibacterium* strains on ruminal fermentation, nutrient digestibility and methane emissions in beef cattle fed a corn grain finishing diet. Animal.

[12] Lettat A, Noziere P, Silberberg M, Morgavi D, Berger C, Martin C (2012). Rumen microbial and fermentation characteristics are affected differently by bacterial probiotic supplementation during induced lactic and subacute acidosis in sheep. BMC Microbiol.

[13] INRA (2007). Nutrition of cattle, sheep and goats: animal needs – feed values.

[14] International_Standarization_Organization. Animal feeding stuffs - Determination of moisture and other volatile matter content. ISO 6496 - AFNOR V18A 1999.

[15] AOAC (2005). Official methods of analysis of AOAC international*.* 16th ed.

[16] van Soest PJ, Robertson JB, Lewis BA (1991). Methods for dietary fiber, neutral detergent fiber, and nonstarch polysaccharides in relation to animal production. J Dairy Sci.

[17] Faisant N, Planchot V, Kozlowski F, Pacouret MP, Colonna P, Champ M (1995). Resistant starch determination adapted to products containing high levels of resistant starch. Sci Aliments.

[18] Guyader J, Eugène M, Meunier B, Doreau M, Morgavi DP, Silberberg M (2015). Additive methane-mitigating effect between linseed oil and nitrate fed to cattle. J Anim Sci.

[19] Pinares-Patino CS, Hunt C, Martin R, West J, Lovejoy P, Waghorn G. Chapter 1: New Zealand ruminant methane measurement Centre, AgResearch, Palmerston North. In: Pinares-Patino CS, Waghorn G, editors. Technical manual on respiration chamber designs. Wellington; 2012. p. 9–28.

[20] Morgavi DP, Boudra H, Jouany JP, Graviou D (2003). Prevention of patulin toxicity on rumen microbial fermentation by SH-containing reducing agents. J Agric Food Chem.

[21] Park GE, Oh HN, Ahn S (2009). Improvement of the ammonia analysis by the phenate method in water and wastewater. B Korean Chem Soc.

[22] Ranilla MJ, Jouany JP, Morgavi DP (2007). Methane production and substrate degradation by rumen microbial communities containing single protozoal species in vitro. Lett Appl Microbiol.

[23] Yu Z, Morrison M (2004). Improved extraction of PCR-quality community DNA from digesta and fecal samples. Biotechniques.

[24] Popova M, Martin C, Eugène M, Mialon MM, Doreau M, Morgavi DP (2011). Effect of fibre- and starch-rich finishing diets on methanogenic Archaea diversity and activity in the rumen of feedlot bulls. Anim Feed Sci Tech.

[25] Ferlay A, Doreau M, Martin C, Chilliard Y (2013). Effects of incremental amounts of extruded linseed on the milk fatty acid composition of dairy cows receiving hay or corn silage. J Dairy Sci.

[26] Stein DR, Allen DT, Perry EB, Bruner JC, Gates KW, Rehberger TG (2006). Effects of feeding propionibacteria to dairy cows on milk yield, milk components, and reproduction. J Dairy Sci.

[27] Vyas D, Alazzeh A, McGinn SM, McAllister TA, Harstad OM, Holo H (2016). Enteric methane emissions in response to ruminal inoculation of *Propionibacterium* strains in beef cattle fed a mixed diet. Anim Prod Sci.

[28] de Ondarza MB, Seymour WM (2008). Effect of propionibacteria supplementation on yield of milk and milk components of dairy cows. Prof Anim Sci.

[29] Francisco CC, Chamberlain CS, Waldner DN, Wettemann RP, Spicer LJ (2002). Propionibacteria fed to dairy cows: effects on energy balance, plasma metabolites and hormones, and reproduction. J Dairy Sci.

[30] Friggens NC, Berg P, Theilgaard P, Korsgaard IR, Ingvartsen KL, Lovendahl P (2007). Breed and parity effects on energy balance profiles through lactation: evidence of genetically driven body energy change. J Dairy Sci.

[31] Negussie E, de Haas Y, Dehareng F, Dewhurst RJ, Dijkstra J, Gengler N (2017). Invited review: large-scale indirect measurements for enteric methane emissions in dairy cattle: a review of proxies and their potential for use in management and breeding decisions. J Dairy Sci.

[32] Yang B, Gao H, Stanton C, Ross RP, Zhang H, Chen YQ (2017). Bacterial conjugated linoleic acid production and their applications. Prog Lipid Res.

[33] Apas AL, Arena ME, Colombo S, Gonzalez SN (2015). Probiotic administration modifies the milk fatty acid profile, intestinal morphology, and intestinal fatty acid profile of goats. J Dairy Sci.

