# Supplementary material for: Bacterial direct-fed microbials fail to reduce methane emissions in primiparous lactating dairy cows
Source: J Anim Sci Biotechnol. 2019 May 2;10:41. doi: 10.1186/s40104-019-0342-9 (PMC6495644; doi:10.1186/s40104-019-0342-9)
Supplement: Supplementary file 1 — Table S1. Primers used in this study. (DOCX 33 kb) [file 40104_2019_342_MOESM1_ESM.docx]

**Additional file 1**

**Table S1.** *Primers used in this study*

| Target gene | Primers | Use | Primers Sequence (5′-3′) | Reference |
| --- | --- | --- | --- | --- |
| *mcrA* methanogen | qmcrA-F | qPCR | TTCGGTGGATCDCARAGRGC | [1] |
|  | qmcrA-R |  | GBARGTCGWAWCCGTAGAATCC | [1] |
| 16S rRNA bacteria | 520f | qPCR | AGCAGCCGCGGTAAT | [2] |
|  | 799r |  | AACAGGATTAGATACCCTG | [2] |
| 16S rRNA *Propionibacterium freudenreichii* | 16SPfsF | qPCR | GTCTCAGTTCGGATTGGGGT | [3] |
|  | 16SPfsR |  | GTTCGGGTGTTACCGACTTTCA | Modified from [3] |
| 16S rRNA *Lactobacillus bulgaricus* | Ldel 7 | qPCR | ACAGATGGATGGAGAGCAGA | [4] |
|  | Lac 2 |  | CCTCTTCGCTCGCCGCTACT | [4] |
| 16S-23S intergenic spacer region *Lactobacillus pentosus* | 16S | qPCR | GCTGGATCACCTCCTTTC | [5] |
|  | LPe |  | GTATTCAACTTATTAGAACG | [5] |

1. Denman SE, Tomkins NW, McSweeney CS: Quantitation and diversity analysis of ruminal methanogenic populations in response to the antimethanogenic compound bromochloromethane. *FEMS Microbiol Ecol* 2007, 62:313-322.

2. Edwards JE, Huws SA, Kim EJ, Kingston-Smith AH: Characterization of the dynamics of initial bacterial colonization of nonconserved forage in the bovine rumen. *FEMS Microbiol Ecol* 2007, 62:323-335.

3. Falentin H, Deutsch SM, Jan G, Loux V, Thierry A, Parayre S, Maillard MB, Dherbecourt J, Cousin FJ, Jardin J, et al: The complete genome of *Propionibacterium freudenreichii* CIRM-BIA1, a hardy actinobacterium with food and probiotic applications. *PLoS One* 2010, 5:e11748.

4. Drisko J, Bischoff B, Giles C, Adelson M, Rao RV, McCallum R: Evaluation of five probiotic products for label claims by DNA extraction and polymerase chain reaction analysis. *Dig Dis Sci* 2005, 50:1113-1117.

5. Berthier F, Ehrlich SD: Rapid species identification within two groups of closely related lactobacilli using PCR primers that target the 16S/23S rRNA spacer region. *FEMS Microbiol Lett* 1998, 161:97-106.
