# Supplementary material for: Bacterial direct-fed microbials fail to reduce methane emissions in primiparous lactating dairy cows
Source: J Anim Sci Biotechnol. 2019 May 2;10:41. doi: 10.1186/s40104-019-0342-9 (PMC6495644; doi:10.1186/s40104-019-0342-9)
Supplement: Supplementary file 2 — Table S2. Ruminal fermentation parameters of lactating cows fed high-starch (HSD) or high-fiber diets (HFD) supplemented with bacterial direct-fed microbials (DFM) Propionibacterium freudenreichii 53 W (PF), Lactobacillus pentosus D31 (LP), and Lactobacillus bulgaricus D1 (LB). (DOCX 33 kb) [file 40104_2019_342_MOESM2_ESM.docx]

**Additional file 2**

**Table S2.** Ruminal fermentation parameters of lactating cows fed High-starch (HSD) or High-fiber diets (HFD) supplemented with bacterial direct-fed microbials (DFM) *Propionibacterium freudenreichii* 53W (PF), *Lactobacillus pentosus* D31 (LP), and *Lactobacillus bulgaricus* D1 (LB)

|  | Treatment | | | | *P* value |
| --- | --- | --- | --- | --- | --- |
| Item | CTL | PF | LP | LB | CTL vs DFM |
| pH |  |  |  |  |  |
| High starch diet | 6.9 | 6.9 | 7.2 | 6.8 | 0.87 |
| High fiber diet | 6.7 | 6.8 | 6.9 | 7.0* | 0.03 |
| High starch diet | 9.5 | 13.1 | 8.1 | 9.2 | 0.71 |
| High fiber diet | 8.5 | 8.5 | 7.6 | 8.6 | 0.80 |
| High starch diet | 86.9 | 84.4 | 69.8 | 94.1 | 0.74 |
| High fiber diet | 92.4 | 93.3 | 91.0 | 86.0 | 0.68 |
| Acetate |  |  |  |  |  |
| High starch diet | 64.7 | 64.6 | 64.7 | 64.5 | 0.97 |
| High fiber diet | 65.0 | 62.3 | 65.5 | 64.2 | 0.42 |
| Propionate |  |  |  |  |  |
| High starch diet | 18.4 | 17.5 | 19.0 | 18.2 | 0.93 |
| High fiber diet | 20.6 | 21.0 | 20.5 | 21.2 | 0.32 |
| Butyrate |  |  |  |  |  |
| High starch diet | 13.0 | 13.9 | 12.2 | 13.3 | 0.94 |
| High fiber diet | 11.9 | 12.8 | 11.3 | 11.8 | 0.95 |
| Isobutyrate |  |  |  |  |  |
| High starch diet | 0.52 | 0.63 | 0.60 | 0.43 | 0.71 |
| High fiber diet | 0.57 | 0.63 | 0.63 | 0.59 | 0.35 |
| Isovalerate |  |  |  |  |  |
| High starch diet | 0.95 | 0.89 | 0.80 | 0.90 | 0.78 |
| High fiber diet | 0.64 | 0.69 | 0.73 | 0.68 | 0.60 |
| Valerate |  |  |  |  |  |
| High starch diet | 1.96 | 1.96 | 1.92 | 2.02 | 0.98 |
| High fiber diet | 0.98 | 1.16 | 1.00 | 1.10 | 0.16 |
| Caproate |  |  |  |  |  |
| High starch diet | 0.56 | 0.61 | 0.78 | 0.71 | 0.46 |
| High fiber diet | 0.34 | 0.38 | 0.36 | 0.38 | 0.52 |
| Acetate/Propionate |  |  |  |  |  |
| High starch diet | 3.66 | 3.76 | 3.52 | 3.69 | 0.99 |
| High fiber diet | 3.18 | 2.90 | 3.22 | 3.04 | 0.31 |

* Significantly (*P* ≤ 0.05) different from CTL group.
