# Supplementary material for: Bacterial direct-fed microbials fail to reduce methane emissions in primiparous lactating dairy cows
Source: J Anim Sci Biotechnol. 2019 May 2;10:41. doi: 10.1186/s40104-019-0342-9 (PMC6495644; doi:10.1186/s40104-019-0342-9)
Supplement: Supplementary file 3 — Table S3. Ruminal concentration of bacteria, archaea, and protozoa (per mL rumen fluid) of lactating cows fed high-starch (HSD) or high-fiber diets (HFD) supplemented with bacterial direct-fed microbials (DFM) Propionibacterium freudenreichii 53 W (PF), Lactobacillus pentosus D31 (LP), and Lactobacillus bulgaricus D1 (LB). (DOCX 31 kb) [file 40104_2019_342_MOESM3_ESM.docx]

**Additional file 3**

**Table S3.** Ruminal concentration of bacteria, archaea, and protozoa (per mL rumen fluid) of lactating cows fed High-starch (HSD) or High-fiber diets (HFD) supplemented with bacterial direct-fed microbials (DFM) *Propionibacterium freudenreichii* 53W (PF), *Lactobacillus pentosus* D31 (LP), and *Lactobacillus bulgaricus* D1 (LB)

|  | Treatment | | | | *P* value |  |
| --- | --- | --- | --- | --- | --- | --- |
|  | CTL^a^ | PF | LP | LB | CTL vs DFM^b^ | |
| Bacterial rrs copies |  |  |  |  |  | |
| High starch diet | 11.60 | 11.38 | 11.62 | 11.52 | 0.32 | |
| High fiber diet | 11.36 | 11.33 | 11.39 | 11.36 | 0.99 | |
| Methanogen mcrA copies |  |  |  |  |  | |
| High starch diet | 9.09 | 8.87 | 9.21 | 8.86 | 0.47 | |
| High fiber diet | 9.17 | 8.94 | 8.99 | 9.02 | 0.20 | |
| Total protozoa |  |  |  |  |  | |
| High starch diet | 5.05 | 5.10 | 4.66 | 4.88 | 0.44 | |
| High fiber diet | 5.04 | 5.02 | 4.94 | 4.93 | 0.42 | |
| Protozoal composition |  |  |  |  |  | |
| Entodiniomorphs(<100 µm) |  |  |  |  |  | |
| High starch diet | 5.02 | 5.08 | 4.62 | 4.86 | 0.43 | |
| High fiber diet | 5.01 | 4.98 | 4.91 | 4.88 | 0.40 | |
| Entodiniomorphs(>100 µm) |  |  |  |  |  | |
| High starch diet | 3.79 | 3.84 | 3.53 | 3.42 | 0.35 | |
| High fiber diet | 3.49 | 3.32 | 2.96 | 3.69 | 0.72 | |
| Total Entodiniomorphs |  |  |  |  |  | |
| High starch diet | 5.05 | 5.10 | 4.65 | 4.87 | 0.43 | |
| High fiber diet | 5.04 | 5.02 | 4.93 | 4.92 | 0.39 | |
| Dasytricha |  |  |  |  |  | |
| High starch diet | 1.75 | 1.39 | 1.04 | 1.93 | 0.72 | |
| High fiber diet | 2.38 | 2.31 | 3.03 | 3.11 | 0.37 | |
| Isotricha |  |  |  |  |  | |
| High starch diet | 2.97 | 1.88 | 2.44 | 2.42 | 0.10 | |
| High fiber diet | 1.29 | 1.82 | 1.24 | 1.13 | 0.88 | |

^a^CTL: control diet without DFM.

^b^*P*-value for control vs all direct-fed microbials (DFM) within each diet
