# Supplementary material for: Bacterial direct-fed microbials fail to reduce methane emissions in primiparous lactating dairy cows
Source: J Anim Sci Biotechnol. 2019 May 2;10:41. doi: 10.1186/s40104-019-0342-9 (PMC6495644; doi:10.1186/s40104-019-0342-9)
Supplement: Supplementary file 4 — Table S4. Major milk fatty acid (FA) composition of cows fed high-starch (HSD) or high-fiber diets (HFD) supplemented with bacterial direct-fed microbials (DFM) Propionibacterium freudenreichii 53 W (PF), Lactobacillus pentosus D31 (LP), and Lactobacillus bulgaricus D1 (LB). (DOCX 69 kb) [file 40104_2019_342_MOESM4_ESM.docx]

**Additional file 4**

**Table S4.** Major^a^ milk fatty acid (FA) composition of cows fed high-starch (HSD) or high-fiber diets (HFD) supplemented with bacterial direct-fed microbials (DFM) *Propionibacterium freudenreichii* 53W (PF), *Lactobacillus pentosus* D31 (LP), and *Lactobacillus bulgaricus* D1 (LB).

|  | Treatment | | | | *P* value |
| --- | --- | --- | --- | --- | --- |
|  | CTL^b^ | PF | LP | LB | CTL vs DFM^c^ |
| **Saturated (SFA)** |  |  |  |  |  |
| 4:0 |  |  |  |  |  |
| High-starch diet | 2.96 | 3.17 | 3.17 | 3.12 | 0.34 |
| High-fiber diet | 3.15 | 2.93 | 3.22 | 2.89 | 0.49 |
| 6:0 |  |  |  |  |  |
| High-starch diet | 2.09 | 2.35 | 2.33 | 2.20 | 0.23 |
| High-fiber diet | 2.33 | 2.14 | 2.27 | 2.10 | 0.16 |
| 8:0 |  |  |  |  |  |
| High-starch diet | 1.23 | 1.36 | 1.37 | 1.26 | 0.42 |
| High-fiber diet | 1.33 | 1.21 | 1.24 | 1.17^¶^ | 0.10 |
| 10:0 |  |  |  |  |  |
| High-starch diet | 2.83 | 3.10 | 3.07 | 2.86 | 0.60 |
| High-fiber diet | 3.02 | 2.80 | 2.67 | 2.65 | 0.19 |
| 12:0 |  |  |  |  |  |
| High-starch diet | 3.25 | 3.55 | 3.65 | 3.28 | 0.57 |
| High-fiber diet | 3.37 | 3.14 | 2.94 | 2.88 | 0.23 |
| 13:0 anteiso |  |  |  |  |  |
| High-starch diet | 0.08 | 0.10 | 0.10 | 0.10 | 0.19 |
| High-fiber diet | 0.07 | 0.06 | 0.07 | 0.07 | 0.47 |
| 14:0 |  |  |  |  |  |
| High-starch diet | 10.9 | 11.3 | 11.8 | 11.2 | 0.49 |
| High-fiber diet | 11.1 | 10.1 | 10.6 | 10.5 | 0.17 |
| 14:0 iso |  |  |  |  |  |
| High-starch diet | 0.07 | 0.07 | 0.08 | 0.06 | 0.76 |
| High-fiber diet | 0.12 | 0.12 | 0.11 | 0.13^¶^ | 0.43 |
| 15:0 |  |  |  |  |  |
| High-starch diet | 1.12 | 1.14 | 1.35 | 1.18 | 0.67 |
| High-fiber diet | 1.43 | 1.40 | 1.32* | 1.53^¶^ | 0.71 |
| 15:0 iso |  |  |  |  |  |
| High-starch diet | 0.22 | 0.22 | 0.23 | 0.22 | 0.83 |
| High-fiber diet | 0.24 | 0.26 | 0.23 | 0.27 | 0.52 |
| 15:0 anteiso |  |  |  |  |  |
| High-starch diet | 0.48 | 0.48 | 0.52 | 0.48 | 0.73 |
| High-fiber diet | 0.55 | 0.50 | 0.54 | 0.60 | 0.95 |
| 16:0 |  |  |  |  |  |
| High-starch diet | 30.6 | 33.4^¶^ | 32.9 | 31.7 | 0.13 |
| High-fiber diet | 35.8 | 33.6 | 34.8 | 33.2^¶^ | 0.09 |
| 16:0 iso |  |  |  |  |  |
| High-starch diet | 0.19 | 0.22 | 0.22 | 0.21 | 0.15 |
| High-fiber diet | 0.31 | 0.32 | 0.29 | 0.33^¶^ | 0.44 |
| 17:0 |  |  |  |  |  |
| High-starch diet | 0.87 | 0.83 | 0.88 | 0.86 | 0.88 |
| High-fiber diet | 0.87 | 0.93* | 0.88 | 0.94* | 0.04 |
| 17:0 iso^d^ |  |  |  |  |  |
| High-starch diet | 0.56 | 0.50 | 0.52 | 0.54 | 0.37 |
| High-fiber diet | 0.46 | 0.52 | 0.48 | 0.51 | 0.22 |
| 17:0 anteiso |  |  |  |  |  |
| High-starch diet | 0.54 | 0.53 | 0.55 | 0.51 | 0.74 |
| High-fiber diet | 0.48 | 0.48 | 0.49 | 0.53 | 0.64 |
| 18:0 |  |  |  |  |  |
| High-starch diet | 7.69 | 7.07 | 6.99 | 7.20 | 0.36 |
| High-fiber diet | 7.28 | 8.31 | 7.32 | 8.19 | 0.20 |
| 18:0 iso |  |  |  |  |  |
| High-starch diet | 0.08 | 0.07 | 0.06 | 0.07 | 0.24 |
| High-fiber diet | 0.08 | 0.10 | 0.10 | 0.10 | 0.14 |
| 20:0 |  |  |  |  |  |
| High-starch diet | 0.09 | 0.09 | 0.08 | 0.10 | 0.87 |
| High-fiber diet | 0.13 | 0.13 | 0.12 | 0.13 | 0.99 |
| 22:0 |  |  |  |  |  |
| High-starch diet | 0.11 | 0.10 | 0.12 | 0.10 | 0.65 |
| High-fiber diet | 0.07 | 0.07 | 0.08 | 0.07 | 0.90 |
| **Monounsaturated (MUFA)** | |  |  |  |  |
| Cis-9 10:1 |  |  |  |  |  |
| High-starch diet | 0.24 | 0.29 | 0.29 | 0.25 | 0.23 |
| High-fiber diet | 0.26 | 0.19^¶^ | 0.25 | 0.21 | 0.19 |
| Cis-9 12:1^e^ |  |  |  |  |  |
| High-starch diet | 0.18 | 0.21 | 0.24 | 0.20 | 0.48 |
| High-fiber diet | 0.18 | 0.17 | 0.15 | 0.17 | 0.31 |
| Cis-9 14:1 |  |  |  |  |  |
| High-starch diet | 1.17 | 1.33 | 1.37 | 1.23 | 0.28 |
| High-fiber diet | 1.06 | 0.70 | 1.01 | 0.84 | 0.31 |
| Cis-6 + 8 + Trans-11 16:1 |  |  |  |  |  |
| High-starch diet | 0.25 | 0.22 | 0.21 | 0.26 | 0.29 |
| High-fiber diet | 0.16 | 0.21 | 0.21 | 0.23^¶^ | 0.08 |
| Cis-9 16:1 |  |  |  |  |  |
| High-starch diet | 1.87 | 1.97 | 1.90 | 1.94 | 0.39 |
| High-fiber diet | 1.92 | 2.03 | 1.90 | 1.99 | 0.63 |
| Cis-9 17:1 |  |  |  |  |  |
| High-starch diet | 0.38 | 0.32^¶^ | 0.31* | 0.36 | 0.08 |
| High-fiber diet | 0.33 | 0.40^¶^ | 0.36 | 0.41^¶^ | <0.10 |
| Cis-9 18:1 |  |  |  |  |  |
| High-starch diet | 20.47 | 17.65 | 16.71^¶^ | 19.36 | 0.12 |
| High-fiber diet | 15.49 | 18.25 | 17.64 | 18.48^¶^ | 0.07 |
| Cis-11 18:1 |  |  |  |  |  |
| High-starch diet | 1.14 | 0.91^¶^ | 0.88^*^ | 1.09 | 0.07 |
| High-fiber diet | 0.79 | 0.93 | 0.94 | 0.97 | 0.18 |
| Cis-12 18:1 |  |  |  |  |  |
| High-starch diet | 0.32 | 0.27 | 0.26^¶^ | 0.31 | 0.15 |
| High-fiber diet | 0.19 | 0.20 | 0.20 | 0.21 | 0.36 |
| Cis-13 18:1 |  |  |  |  |  |
| High-starch diet | 0.15 | 0.12* | 0.10* | 0.15 | 0.01 |
| High-fiber diet | 0.10 | 0.12 | 0.12 | 0.11 | 0.24 |
| Cis-15 18:1^f^ |  |  |  |  |  |
| High-starch diet | 0.17 | 0.16 | 0.16 | 0.18 | 0.94 |
| High-fiber diet | 0.16 | 0.18 | 0.16 | 0.17 | 0.34 |
| Trans-6 + 8 18:1 |  |  |  |  |  |
| High-starch diet | 0.18 | 0.17 | 0.20 | 0.18 | 0.83 |
| High-fiber diet | 0.11 | 0.12 | 0.11 | 0.12 | 0.67 |
| Trans-9 18:1 |  |  |  |  |  |
| High-starch diet | 0.18 | 0.16 | 0.16 | 0.16 | 0.15 |
| High-fiber diet | 0.13 | 0.14 | 0.13 | 0.14 | 0.32 |
| Trans-10 18:1 |  |  |  |  |  |
| High-starch diet | 0.53 | 0.54 | 0.80 | 0.60 | 0.57 |
| High-fiber diet | 0.16 | 0.14 | 0.15 | 0.16 | 0.64 |
| Trans-11 18:1 |  |  |  |  |  |
| High-starch diet | 0.83 | 0.72 | 0.80 | 0.72 | 0.44 |
| High-fiber diet | 0.89 | 1.00 | 0.94 | 1.04 | 0.32 |
| Trans-12 18:1 |  |  |  |  |  |
| High-starch diet | 0.28 | 0.26 | 0.27 | 0.27 | 0.57 |
| High-fiber diet | 0.18 | 0.19 | 0.18 | 0.20 | 0.72 |
| Trans-13 18:1 |  |  |  |  |  |
| High-starch diet | 0.35 | 0.33 | 0.39 | 0.31 | 0.90 |
| High-fiber diet | 0.38 | 0.31 | 0.32 | 0.30 | <0.10 |
| Trans-16 + cis-14 18:1 |  |  |  |  |  |
| High-starch diet | 0.24 | 0.22 | 0.22 | 0.24 | 0.59 |
| High-fiber diet | 0.24 | 0.25 | 0.23 | 0.24 | 0.98 |
| **Polyunsaturated (PUFA)** |  |  |  |  |  |
| Cis-9 cis-12 18:2 |  |  |  |  |  |
| High-starch diet | 2.04 | 1.86 | 1.83 | 1.99 | 0.45 |
| High-fiber diet | 1.44 | 1.58 | 1.60 | 1.54 | 0.25 |
| Cis-9 trans-13 18:2 |  |  |  |  |  |
| High-starch diet | 0.16 | 0.14 | 0.14 | 0.16 | 0.30 |
| High-fiber diet | 0.14 | 0.16 | 0.14 | 0.14 | 0.61 |
| Cis-9 trans-12 18:2^g^ |  |  |  |  |  |
| High-starch diet | 0.13 | 0.12 | 0.12 | 0.14 | 0.96 |
| High-fiber diet | 0.14 | 0.12 | 0.14 | 0.14 | 0.90 |
| Trans-11 cis-15 18:2 |  |  |  |  |  |
| High-starch diet | 0.08 | 0.07 | 0.07 | 0.09 | 0.75 |
| High-fiber diet | 0.06 | 0.08^*^ | 0.07 | 0.07 | 0.10 |
| C18:3n-3 |  |  |  |  |  |
| High-starch diet | 0.25 | 0.20 | 0.23 | 0.25 | 0.32 |
| High-fiber diet | 0.53 | 0.52 | 0.58 | 0.56 | 0.33 |
| C20:3n-6 |  |  |  |  |  |
| High-starch diet | 0.08 | 0.09 | 0.10 | 0.09 | 0.49 |
| High-fiber diet | 0.07 | 0.07 | 0.07 | 0.06 | 0.53 |
| C20:4n-6 |  |  |  |  |  |
| High-starch diet | 0.16 | 0.15 | 0.15 | 0.16 | 0.88 |
| High-fiber diet | 0.12 | 0.14 | 0.12 | 0.14 | 0.35 |
| C22:5n-3 |  |  |  |  |  |
| High-starch diet | 0.10 | 0.10 | 0.11 | 0.10 | 0.77 |
| High-fiber diet | 0.17 | 0.20 | 0.17 | 0.20 | 0.36 |
| Cis-9 trans-11 Conjugated 18:2 (CLA)^h^ |  |  |  |  |  |
| High-starch diet | 0.41 | 0.37 | 0.39 | 0.39 | 0.62 |
| High-fiber diet | 0.42 | 0.43 | 0.43 | 0.46 | 0.66 |
|  |  |  |  |  |  |
| Σ SFA |  |  |  |  |  |
| High-starch diet | 65.7 | 69.5^¶^ | 69.9^¶^ | 67.0 | 0.10 |
| High-fiber diet | 72.1 | 69.0 | 69.6 | 68.6^¶^ | 0.07 |
| Σ Odd- & branched-chain FA |  |  |  |  |  |
| High-starch diet | 3.89 | 3.92 | 4.28 | 3.96 | 0.67 |
| High-fiber diet | 4.41 | 4.41 | 4.27 | 4.78^¶^ | 0.62 |
| Σ MUFA |  |  |  |  |  |
| High-starch diet | 28.9 | 25.7^¶^ | 25.1 | 27.7 | 0.11 |
| High-fiber diet | 22.6 | 25.4 | 24.9 | 25.9^¶^ | 0.07 |
| Σ trans FA |  |  |  |  |  |
| High-starch diet | 3.53 | 3.15 | 3.65 | 3.35 | 0.72 |
| High-fiber diet | 2.67 | 2.77 | 2.64 | 2.79 | 0.60 |
| Σ PUFA |  |  |  |  |  |
| High-starch diet | 3.76 | 3.33 | 3.43 | 3.70 | 0.33 |
| High-fiber diet | 3.39 | 3.68 | 3.60 | 3.60 | 0.26 |
| Σ CLA |  |  |  |  |  |
| High-starch diet | 0.47 | 0.40 | 0.44 | 0.41 | 0.31 |
| High-fiber diet | 0.46 | 0.49 | 0.48 | 0.51 | 0.42 |
| Σ n-3 FA |  |  |  |  |  |
| High-starch diet | 0.40 | 0.34 | 0.38 | 0.41 | 0.44 |
| High-fiber diet | 0.79 | 0.85 | 0.82 | 0.84 | 0.42 |
| Σ n-6 FA |  |  |  |  |  |
| High-starch diet | 2.39 | 2.19 | 2.18 | 2.37 | 0.49 |
| High-fiber diet | 1.72 | 1.89 | 1.88 | 1.83 | 0.27 |
| trans-10 18:1/trans-11 18:1 |  |  |  |  |  |
| High-starch diet | 0.66 | 0.73 | 0.97 | 0.94 | 0.49 |
| High-fiber diet | 0.18 | 0.15 | 0.16 | 0.16 | 0.30 |

^a^ Major FA: ≥ 0.10 g/100 g of FA

^b^CTL: control diet without DFM.

^c^*P*-value for control vs all direct-fed microbials (DFM) within each diet

^d^Co-eluted with trans-9 16:1

^e^ Co-eluted with 13:0

^f^ Co-eluted with 19:0

^g^ Co-eluted with cis-9 trans-14 18:2 and cis-16 18:1

^h^ Co-eluted with trans-7 cis-9 and trans-8 cis-10 CLA

* Significantly (*P* ≤ 0.05) different from CTL group. ^¶^ Differs (*P* ≤ 0.10) different from CTL group
